# The effects of six weeks of supplementation with multi-ingredient performance supplements and resistance training on anabolic hormones, body composition, strength, and power in resistance-trained men

**DOI:** 10.1186/1550-2783-9-49

**Published:** 2012-11-15

**Authors:** Michael J Ormsbee, W Kyle Mandler, D David Thomas, Emery G Ward, Amber W Kinsey, Emily Simonavice, Lynn B Panton, Jeong-Su Kim

**Affiliations:** 1 Department of Nutrition, Food and Exercise Sciences, Institute of Sports Science and Medicine, The Florida State University, 120 Convocation Way, 430 Sandels Building, Tallahassee, FL, 32306-1493, USA; 2 Department of Kinesiology, Georgia College and State University, Miledgeville, GA, 31061, USA

**Keywords:** Sports nutrition, Wingate test, Body composition, Maximal strength, Testosterone

## Abstract

**Background:**

Resistance training (RT) enhances muscle protein synthesis and hypertrophy while increasing strength and power. Some multi-ingredient performance supplements (MIPS) have been shown to augment the physiological improvements associated with RT. The purpose of this study was to investigate the impact of specific pre- and post-workout MIPS on anabolic hormones, body composition, muscle strength, and power in resistance-trained men participating in a periodized RT program.

**Methods:**

Twenty-four ( mean ± SE; 24.0 ± 0.9 years; 180.5 ± 5.8 cm; 83.7 ± 0.5 kg) resistance-trained men completed 6 wks of periodized RT (3x/wk). Participants were assigned to one of two groups based upon maximal voluntary contraction of the quadriceps (Biodex) to lean mass (LM) ratio. Group 1 (n = 13; MIPS) consumed one serving of NO-Shotgun® (whey protein, casein protein, branched-chain amino acids, creatine, beta alanine, and caffeine) before each workout and one serving of NO-Synthesize® (whey protein, casein protein, branched-chain amino acids, creatine, and beta alanine; Vital Pharmaceuticals, Inc., Davie, FL) immediately after each workout and on non-RT days. Group 2 (n = 11; Placebo; PLA) consumed a flavor-matched isocaloric maltodextrin placebo. Serum insulin-like growth factor 1, human growth hormone, testosterone, body composition (DXA), circumferences, 1-repetition maximal strength (1RM) of the upper (chest press) and lower body (leg press), and anaerobic power (Wingate test) were assessed before and after the intervention. Statistical analysis included a 2 × 2 (group x time) ANOVA with repeated measures. Tukey LSD post hoc tests were used to examine pairwise differences. Significance was set at p < 0.05.

**Results:**

There was a main time effect (p = 0.035) for testosterone to increase, but no differences between groups were observed. There were no differences in the other blood hormones. Group x time interactions were observed for LM (MIPS: PRE, 62.9 ± 2.1 to POST, 65.7 ± 2.0 vs. PLA: PRE, 63.5 ± 2.3 to POST, 64.8 ± 2.5 kg; p = 0.017). Only a main effect of time was noted for circumference measures. Both groups increased upper and lower body 1RM strength to a similar degree. MIPS significantly increased peak anaerobic power (PRE, 932.7 ± 172.5 W vs. POST, 1119.2 ± 183.8 W, p = 0.002) while PLA remained unchanged (PRE, 974.4 ± 44.1 W vs. POST, 1033.7 ± 48.6 W, p = 0.166).

**Conclusion:**

Consumption of MIPS during the course of a periodized RT program facilitated training-induced improvement in LM in trained males, whereas the consumption of PLA did not. MIPS improved measures of anaerobic power while PLA did not.

## Background

Multi-ingredient performance supplements (MIPS) intended for consumption in close proximity to resistance exercise are extremely popular among young males [1,2] and athletes [3]. The composition of MIPS vary widely, but the principle ingredients generally include creatine monohydrate, caffeine, beta alanine, the branched chain amino acids (BCAAs) leucine, isoleucine, and valine, as well as L-citrulline, and L-arginine. Most of these ingredients have been shown singularly [4-10] and in combination [11-14] to exert ergogenic effects during aerobic and anaerobic exercise or facilitate muscle hypertrophy over the course of a resistance training (RT) period in untrained participants.

Claims about effectiveness and ergogenic enhancements provided by MIPS are often not supported by empirical data and worse, frequently reflect poor understanding or even a misappropriation of the underlying science. Accordingly, it is of importance to consumers and researchers that MIPS be evaluated in double-blinded, placebo-controlled trials. While there is a considerable body of research on the individual effects of creatine, caffeine, beta alanine and protein/amino acid consumption in proximity to exercise [4,6,9,15-20], there is a paucity of data regarding the combined effect of these ingredients on exercise performance with RT [14,21,22]. The limited evidence available suggests that MIPS products of this general composition may offer an advantage for those wishing to increase muscle mass and strength.

Smith et al. supplemented twenty-four moderately-trained recreational athletes with a pre-workout supplement (Game Time®, Corr-Jensen Laboratories Inc., Aurora, CO), containing 18 g of a proprietary blend including whey protein, cordyceps sinensis, creatine monohydrate, citrulline, ginseng, and caffeine [11,12]. Participants in this study performed nine high intensity interval run training sessions over 3 weeks. Participants consumed Game Time® or placebo 30 minutes prior to each training session. In contrast to the placebo group, the supplemented participants demonstrated a significantly higher training volume (distance to exhaustion per session) and tended to perform better in measures of anaerobic running capacity (Game Time®: 11.6% versus placebo). Additionally, lean mass (LM) increased from 54.2 ±3.5 kg to 55.4 ± 3.7 kg (p = 0.035) in the Game Time® group and remained unchanged in the placebo group, while there were no significant changes for either group in percent body fat [12].

There has been surprisingly little investigation into the efficacy of these supplements in individuals that are already resistance-trained. Schmitz et al. [22] provided two types of MIPS containing similar creatine, carbohydrate, and protein profiles, but varying in some proprietary ingredients, for consumption immediately before and during RT to men who had been resistance training regularly for at least two years. Following 9 weeks of 4 days/week progressive RT, both groups demonstrated improvements in chest press one repetition maximum (1RM) (MIPS: +19.8% vs. Comparator Product +15.3% p < 0.019 ), and LM (MIPS: + 2.4% vs. Comparator Product +0.27%, p < 0.049 ). However, without a placebo group, it is difficult to say what proportion of these improvements was induced by RT alone.

Shelmadine et al. [14] and Spillane et al. [21] have published data that are distinctly relevant to the current study. These groups both examined the effects of 28 days of MIPS during identical RT programs in untrained men. Shelmadine used the commercially available pre-workout supplement NO-Shotgun® (SHOT, Vital Pharmaceuticals, Davie FL), containing whey protein, caffeine, creatine, beta alanine, BCAAs, and L-arginine with 18 untrained males and compared it to an isocaloric placebo [14]. Maximal 1RM for upper body strength improved for both groups, but more so for MIPS (MIPS: +8.82 ± 1.78% vs. placebo: +0.73 ± 2.30%, p = 0.003), while there were no significant differences in improvements in 1RM lower body strength (MIPS: +18.4 ± 1.91% vs. placebo: +11.99 ± 2.79%, p = 0.10). MIPS also increased fat free mass greater than placebo (MIPS: +4.75 ± 0.50% vs. PL: +1.69 ± 0.54%, p = 0.001). Spillane et al. [21] used SHOT in the same pre-workout manner as Shelmadine et al., but NO-Synthesize® (SYNTH, Vital Pharmaceuticals, Davie FL) was also consumed immediately post-RT and upon waking on non-training days in untrained men. Participants in both the SHOT/SYNTH and placebo groups improved body composition and strength as a result of training, however, the SHOT/SYNTH group had greater increases in fat free mass (p = 0.03), upper (p = 0.02) and lower body (p = 0.04) 1RM strength.

While the findings of Shelmadine [14] and Spillane [21] are promising, especially in regards to significant increases in muscle mass with MIPS use, assumptions must be made to draw conclusions about populations other than untrained males. To date, there have been no investigations of the effectiveness of MIPS taken before and after RT and on non-training days with resistance-trained male participants over the course of an extended (six-week) RT program. Therefore, the aim of this study was to determine the efficacy of pre- and post-RT supplementation with MIPS on anabolic hormones, body composition, muscle strength, and power in resistance-trained men participating in a six-week periodized RT program. We hypothesized that pre- and post-exercise intake of MIPS during a six-week RT program would increase the concentrations of anabolic hormones and enhance gains in muscle mass, strength, and power more than PLA in resistance-trained men.

## Methods

### Participants

Twenty-four resistance-trained (mean ± SE, 5.3 ± 3.5 years of resistance-training for at least twice per week) male participants (age, 24.0 ± 0.9 years; height, 180.5 ± 5.8 cm; body mass, 83.7 ± 0.5 kg; body mass index, BMI, 25.5 ± 2.2 kg/m^2^) completed this study. Participants were nonsmokers and did not have hypertension (blood pressure >140/90), uncontrolled cholesterol/blood lipid levels or take prescription cholesterol medication. They also did not have diagnosed cardiovascular disease, stroke, diabetes, thyroid or kidney dysfunction, or have any musculoskeletal complications that would impede them from performing RT. Individuals who were currently consuming other workout supplements or ergogenic aids were instructed to immediately stop consumption and complete a four-week washout period before entering the study. Individuals consuming anabolic steroids were excluded. All procedures involving human subjects were approved by the Florida State University Human Subjects Institutional Review Board in accordance with the Helsinki Declaration, and written informed consent was obtained prior to participation.

### Experimental design

This study used a stratified, randomized, longitudinal, double-blind design with placebo control. Following the initial collection of pre-testing data and before the start of training, participants were placed into MIPS (n = 13) or placebo (PLA, n = 11) groups. Stratification was based on the ratio of initial maximal voluntary contraction (MVC, isometric 60° knee extension) to LM. Following pre-testing data collection, participants began a periodized six-week resistance training program under direct supervision of research personnel. Participants consumed one 21 g serving of NO-Shotgun® (SHOT; ~72 kcals; 18 g protein; 9.7 g protein hydrolysate matrix including BCAAs; 8.06 g muscle volumizing and power/speed/strength matrix that includes multiple forms of creatine and beta alanine; 376 mg of Redline®energy matrix including caffeine; Vital Pharmaceuticals, Inc., Davie, FL) or isocaloric maltodextrin PLA 15 minutes prior to exercise. Upon the conclusion of each training session, participants immediately consumed one 21 g serving of NO-Synthesize® (SYNTH; ~82 kcals; 20 g protein; 9.7 g protein hydrolysate matrix including BCAAs; 9.4 g muscle volumizing and power/speed/strength matrix that includes multiple forms of creatine and beta alanine; Vital Pharmaceuticals, Inc., Davie, FL) or PLA. Research personnel watched as each participant consumed the supplement on all training days. In addition, participants were given single servings of SYNTH or PLA to consume on non-training days. Laboratory testing took place only before and after the six-week intervention. Participants returned for post-testing at least 36 hours following the final training session in order to minimize the effects of delayed onset muscle soreness and post-exercise reduced maximal torque [23] on testing data, as well as to ensure that any changes were due to chronic training and supplementation rather than acute changes from the final RT session. Participants continued to consume a serving of SYNTH (1x/day) after training had ended until the day of (but not including) post-testing.

### Resistance training protocol

For the duration of the study, three sets of each exercise were completed as a percentage of baseline 1RM. For the first two weeks, participants completed 10 repetitions at 70-75% of 1RM. For weeks three and four, resistance was increased to six repetitions at 80-85% 1RM. For the final two weeks, participants completed four repetitions per set at 85-90% of 1RM. Each major muscle group was trained once per week using at least one exercise. The six-week training program was designed to target every major muscle group in a three-day split and was modified from previously published research [24,25]. The exercises for day one, designed to work the biceps, triceps, and shoulders were performed in the following order: shoulder military press, dumbbell incline biceps curl, cable overhead French press, straight bar curls, cable triceps press down, and dumbbell reverse fly. The exercises for day two, designed to work the muscles of the legs and core, were (in order): leg press (LP), straight leg dead lift, dumbbell lunge, leg curls, standing calf raises, abdominal crunch, and core planks. The third and final day of the rotation was designed to work the muscles of the chest and back with the following exercises (in order): flat chest press (CP), cable pull down, incline CP, cable low row (neutral grip), dumbbell chest flys, and dumbbell shrugs. Three sets of each exercise were performed for prescribed number of repetitions or to failure, whichever came first, with resting times of 60–90 seconds between sets. If a participant was unable to perform the prescribed weight for an exercise, the weight was adjusted to yield failure at or near the specified number of repetitions. The emphasis placed on consistent lifting form in this study, coupled with researcher supervision from certified personal trainers through the National Strength and Conditioning Association (NSCA), helped ensure full participant compliance with training as well as reduce variability due to inter-subject differences or deficiencies in form.

### Testing sessions

Laboratory testing was completed on two occasions. The first visit was prior to the intervention and the second visit was after the six-week intervention. Pre- and post-testing laboratory visits were identical and each measurement was taken by the same investigator at both visits. Participants arrived at the laboratory following an eight-hour overnight fast and had heart rate (60 seconds; radial pulse) and blood pressure (auscultatory method) measured [26] after sitting quietly for five minutes. Each measurement was taken twice and the average was recorded. The following measurements were completed (in order): blood measures, body composition, isokinetic and isometric strength, Wingate, and maximal strength for every participant.

### Blood measures

Blood samples (~10 ml) were drawn via venipuncture from the median cubital or cephalic vein in the antecubital space of the forearm into vacutainer tubes with no preservative (Becton Dickinson, Franklin Lakes, NJ). Serum samples were allowed to clot at room temperature and then stored on ice until centrifuging at 3500 rpm at 4°C for 15 minutes (IEC CL3R Multispeed Centrifuge, Thermo Electron Corporation, Needham Heights, Massachusetts). Aliquots of 300 μL each were transferred into microtubes and frozen at −80 degrees Celsius for later analysis of insulin-like growth factor-1 (IGF-1), human Growth Hormone (hGH), and testosterone using commercially available ELISA kits (R&D Systems, Minneapolis, MN, USA). Intra-assay coefficient of variability was 4.5%, 8.1%, and 15.2% for IGF-1, hGH, and testosterone, respectively. Following blood collection, participants consumed one eight ounce box of apple juice.

### Body composition

Body mass and height (SECA, Hamburg, Germany) were recorded. All measurements were taken with shoes removed wearing only underwear. Body composition was measured using dual-energy x-ray absorptiometry (DXA; GE Lunar iDXA; General Electric Company, Fairfield, Connecticut) with participants in the supine position according to the manufacturer’s instructions. Results were analyzed with enCORE Software, version 11.0 (GE Lunar). The coefficient of variation (CV) for the total body lean and fat tissue were 1.5% and 1.9%, respectively, based on the three repeated measures of a subset of 10 participants.

Circumference measurements of the upper arm, chest, gluteals, and thigh were taken using a measuring tape with strain gauge (Creative Health Products, Ann Arbor, Michigan) and the participant wearing only exercise shorts. For the chest measurement, the tape was run horizontally across the nipples and around the back, and the participant was instructed to exhale fully. For the upper arm measurement, participants were instructed to raise the dominant arm to shoulder height and contract the biceps brachii maximally until the measurement was completed. The measurement was taken at the thickest part of the contracting biceps brachii. The gluteal measurement was taken around the widest part with the participant standing with his feet together. The thigh measurement was taken while the participant stood with the heel of the dominant leg placed on the toes of the opposite foot. The measurement was then taken at the widest part of the dominant leg. A measurement from the top of the patella to the point of circumference measurement was made and recorded to be repeated in the post-test. All measurements were taken by the same researcher on pre- and post-testing laboratory visits.

### Isokinetic and isometric strength

The order of performance testing was uniform for each participant for both laboratory visits. Participants were placed in the upright seated position on a Biodex System 3 (Biodex Medical Systems, Shirley, New York). The seat height and position were adjusted in order to align the instrument’s axis of rotation with that of the participant’s dominant knee. Participants were instructed to cross their arms over their chests, but not to grab the restraints. Isokinetic 30°sec^-1^ and 60°sec^-1^ unilateral knee extension/flexion tests were conducted. Five repetitions of consecutive maximal extension and flexion were performed during each test, with a one minute rest interval between tests. Following the isokinetic tests, a 60° isometric knee extension/flexion test was performed. This test involves three maximal extension and flexion exertion against an immovable arm, with 10 second rest periods between exertions. Continuous verbal encouragement was provided by the research team throughout the duration of all tests. Criterion measures were peak and average torque for each repetition.

### Wingate test

Anaerobic capacity was measured using a Wingate test [27] on a plate loaded and friction braked Monark Ergomedic 874-E (Monark Exercise AB, Vansbro, Sweden) cycle ergometer. Resistance was set as 7.5% of body mass (kg). Each participant was fitted to the ergometer by adjusting the seat height to ensure 5-10° of knee flexion at the bottom of the pedal stroke. The participant performed a two-minute warm-up at 75 rpm with only the resistance added by the weight basket (0.5 kg), with two brief (~10 seconds) bouts of practice sprinting. Following the warm-up period, a five-second countdown period was begun where the participant maximized revolutions per minute. When the participant was cycling at full speed, the resistance was added and the 30-second test timer was started. Throughout the test the participants were given verbal encouragement to work at the highest possible effort and to be aware of the time remaining. At the end of the 30-second test period, the resistance was removed and the participant was instructed to cycle slowly for at least two minutes to cool down. Video of the exercise bout was recorded (Pentax Optio W90, Pentax Imaging Company, Golden, Colorado) and later analyzed to determine total revolutions (R_total_) and peak revolutions (R_max_). The exercise was broken down into five-second intervals (i.e. 0–5 seconds, 5–10 seconds, 10–15 seconds, etc.) and R_max_ was defined as the maximal number of revolutions achieved during each interval. From these values, total work (W) was calculated as (*r* * *R*_*total*_), where *r* is the resistance in kg and R_total_ is the total number of revolutions completed in the 30-second testing period. Peak anaerobic power was calculated as $\left(\frac{r*6m*R\text{max}}{5\;\text{seconds}\;}\right)$, where *R*_*max*_ is the number of revolutions completed in the first five seconds of the test and 6m corresponds to the distance traversed by the flywheel in one revolution (6 meters). Mean anaerobic power was calculated as $\left(\frac{W}{30\;\text{seconds}}\right)$. Fatigue Index was calculated as the ratio of the minimum number of revolutions (R_min_) to R_max_.

### One Repetition Maximum (1RM) Strength

After laboratory pre-testing, but prior to the first training session, participants reported to the training location for the determination of 1RM in the CP and 45° LP exercises. For the purposes of this study, 1RM is defined as the maximum weight an individual is able to perform on a given exercise, with good form, through the full range of motion and was administered according to the NSCA guidelines [28]. Briefly, a warm up with a low resistance and five to 10 repetitions was followed by one minute of rest. A second warm up load was estimated to allow the subject to complete three to five repetitions. Following a two-minute rest period, weight was gradually increased by five to 10% for CP, or 10 to 20% for LP for a single repetition, followed by a two-minute rest period. Weight was increased gradually until a failed attempt or proper form was not maintained. Upon failure, weight was reduced by 2.5-5% for CP, or 5-10% for LP and the participant made another, final attempt after a four-minute rest period. The maximum weight successfully lifted once was recorded as the 1RM for that exercise.

The form cues used for the 1RM and training sessions for each exercise did not differ. For the CP, the participant was to lie flat on the bench with the eyes approximately at the level of the bar as it rests in the rack. The participant was to grasp the bar so that the wrists were situated directly above the elbows for the duration of each repetition. The participant’s back maintained contact with the bench at all times, and did not become unnaturally arched. The participant’s feet remained flat on the floor and the heels did not rise during the exercise. The bar was lowered until the upper arms were parallel with the floor, and the elbows were flexed at approximately 90°, at which point the bar was pressed back to full extension. For the LP, feet were placed on the push plate so that they were just wider than shoulder width and the knees were flexed to approximately 90°. The plate was lowered until the tops of the thighs were just touching the chest, at which point it was pressed out to full extension.

### Nutritional intake and supplementation protocol

After the pre-testing session and at the end of the study, participants were required to complete a three-day food and activity log. Compliance was low for the food logs; only eight participants successfully completed both pre and post training food logs.

Following the completion of all pre-testing, the RT program began. The assigned pre-workout MIPS or PLA was consumed under the supervision of certified research staff 15 minutes prior to the beginning of RT. During this time, a light warm-up on the cardiovascular exercise machine of choice was performed. Immediately upon the completion of each training session, the post-workout MIPS or PLA was consumed. A single serving Ziploc® bag of MIPS or PLA was given to each participant to consume on non-training days. To ensure compliance, these (empty) bags were returned before the subsequent training session and recorded by research personnel. Upon completion of the training sessions, the participants reported back to the laboratory 36 hours following the last RT bout for post-testing, identical to that of the pre-testing visit.

### Statistical analysis

Descriptive data were generated for all variables and expressed as mean ± standard error. A two (group) × two (time) analysis of variance (ANOVA) with repeated measures was used to analyze body composition, strength, power, and hormone data. Tukey LSD post hoc tests were used to examine pairwise differences. Significance was set at p < 0.05. A one-way ANOVA was used for baseline comparisons between groups and volume data. PASW Statistics for Windows version 18.0.0 (International Business Machines Corporation, Armonk, New York, United States) and Statistica (Statsoft, Tulsa, Oklahoma, USA) software were used to perform the analyses.

## Results

No significant differences were noted between groups in any variable before training. There were no differences in total training volume (weight x successful repetitions × sets) between groups (MIPS: 26,583 ± 1,359 kg vs. PLA: 24,200 ± 1,519 kg, p = 0.25). When the values were adjusted for lean mass there were still no differences (MIPS: 400 ± 15 kg vs. PLA: 385 ± 17 kg, p = 0.50).

### Blood measures

No main effects of time or group x time were noted in serum concentrations of IGF-1 or hGH for either group. A main time effect (p = 0.035) was noted for testosterone to increase, but no differences between groups were observed. There were no differences between any hormone variable at the beginning of RT (Table 1).

**Table 1:** **Average serum concentrations of testosterone, human growth hormone (hGh), and insulin-like growth factor-1 (IGF-1**)

**Variable**	**Group**	**PRE**	**POST**	**time**	**group × time**
Testosterone	MIPS (n = 11)	40.2 ± 12.9	58.3 ± 11.5	p = 0.035	p = 0.881
(ng/mL)	PLA (n = 7)	38.9 ± 10.3	54.9 ± 12.4		
hGH	MIPS (n = 12)	113.3 ± 21.0	119.9 ± 35.3	p = 0.510	p = 0.376
(pg/mL)	PLA (n = 7)	71.9 ± 20.6	64.5 ± 13.1		
IGF −1	MIPS (n = 11)	173.2 ± 7.5	181.9 ± 10.5	p = 0.768	p = 0.283
(ng/mL)	PLA (n = 10)	152.9 ± 14.9	147.5 ± 28.4		

A main time effect was observed for both groups to improve serum testosterone, with no difference between groups. Values are presented as means ± SE. Sample size between each analyte is slightly different due technical difficulties with certain samples.

### Body composition

**Figure 1:**
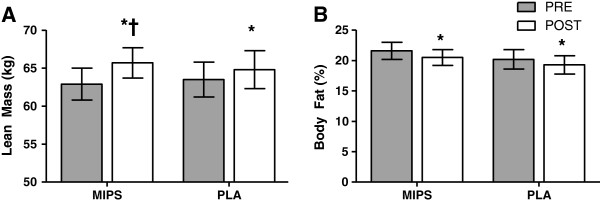
**Lean Mass (kg) and Body Fat percentage before and after six weeks of resistance training and supplementation with multi ingredient performance supplement (MIPS, n = 13) or placebo (PLA, n = 11).** † Indicates group × time effect (p = 0.017). * Indicates main time effect (p = 0.001). Bars are means ± SE.

Significant (p = 0.017) group x time interactions were observed for LM but not for any fat measures. MIPS increased LM by 4.7% (PRE, 62.9 ± 8.8 kg vs. POST, 65.7 ± 8.8 kg, p < 0.001). No significant changes were observed in LM in the PLA group (PRE, 63.5 ± 5.2 kg vs. POST, 64.7 ± 5.9 kg, p = 0.63) group over time with training, although there was a trend for increases in LM (p = 0.085). Both groups demonstrated a main time effect (p = 0.003) for percent body fat (%BF), but no changes were observed in FM (kg). Post-hoc analysis revealed that the MIPS decreased %BF from 21.6 ± 1.4% to 20.5 ± 1.3% (p = 0.004). There was no significant decrease in overall FM. There were no significant changes in fat variables for the PLA group (Figure 1).

### Circumferences

Circumferences of the upper arm, chest, thigh and gluteals were measured pre- and post- training. There were no group x time interactions for any variable. Time effects were observed in chest (p = 0.005), arm (p = 0.001), and gluteals (p = 0.004). Post-hoc analysis indicated that the MIPS group increased arm circumference by 2.2% (PRE, 37.6 ± 0.8 cm vs. POST, 38.5 ± 0.7 cm, p = 0.002) and thigh by 2.5% (PRE, 55.1 ± 1.2 cm vs. POST, 56.6 ± 1.5 cm, p = 0.021). Likewise, the PLA group increased arm circumference by 2.6% (PRE, 36.8 ± 0.90 cm vs. POST, 37.8 ± 0.9 cm, p = 0.001). There were no other significant changes in circumference for either group.

### Isokinetic and isometric strength

There were no group x time interactions observed for any isokinetic variable. Time effects were observed for 30°sec^-1^ extension average power (p = 0.02), 30°sec^-1^ flexion average power (p = 0.01), 30°sec^-1^ agonist/antagonist ratio (p = 0.03). For 60°sec^-1^ extension, time effects were observed for average power (p =0.02) and maximum repetition total work (p = 0.03). For 60°sec^-1^ flexion, time effects were noted for peak power (p = 0.02), maximum repetition total work (p = 0.03), average power (p = 0.004), and average peak torque (p = 0.02).

Post hoc analysis revealed that the MIPS group had no change in relative peak torque (PRE, 254.5 ± 16.5 N-M·kg^-1^ vs. POST, 245.9 ± 12.2 N-M·kg^-1^, p = 0.09) during 30°sec^-1^ extension, however, average power increased 6.2% (PRE, 72.1 ± 3.7 W vs. POST, 76.9 ± 3.6 W, p = 0.02) and acceleration time decreased 52.2% (PRE, 29.2 ± 3.9 ms vs. POST, 19.2 ± 1.9 ms, p = 0.03). During 60°sec^-1^ flexion MIPS peak torque increased 14.5% (PRE, 108.7 ± 4.6 N·M vs. POST, 121.0 ± 6.5 N·M, p = 0.048), maximum repetition total work increased 15.2% (PRE, 103.6 ± 6.9 J vs. POST, 122.1 ± 8.3 J, p = 0.032), and average power increased 13.3% (PRE, 68.8 ± 3.0 W vs. POST 79.5 ± 75.5 W, p = 0.028). There were trends during 60°sec^-1^ extension for an increase in MIPS maximum repetition total work (p = 0.053) and average peak torque (p = 0.052). There was also a trend for improved agonist/antagonist ratio during 30°sec^-1^ isokinetic exercise (p = 0.053).

The PLA group increased average power 17.1% (PRE, 40.6 ± 2.7 W vs. POST, 49.0 ± 2.1 W, p = 0.002) during 30°sec^-1^ flexion, decreased deceleration time 49.1% (PRE, 261.0 ± 0.6 ms vs. POST, 175.0 ± 38.0 ms, p = 0.03), and improved average peak torque 9.6% (PRE, 115.3 ± 6.7 N·M vs. POST, 127.5 ± 6.1 N·M, p = 0.03). There were trends for improvement in average power (p = 0.058) and average peak torque (p = 0.065) during 30°sec^-1^ flexion.

Group x time interactions were observed for relative average peak torque during isometric flexion (p = 0.03). There were also similar trends during isometric flexion for average peak torque (p = 0.053) and relative peak torque (p = 0.057).

Post hoc analysis revealed that there were no changes in any isometric variables for the MIPS group. However, the PLA group improved peak torque by 12.7% (PRE, 123.6 ± 8.1 N·M vs. POST, 141.5 ± 6.9 N·M, p = 0.03), and average peak torque by 12.2% (PRE, 114.2 ± 8.2 N·M vs. POST, 130.9 ± 6.3 N·M, p = 0.047). There was also a trend for improvement in relative peak torque in the PLA group (p = 0.053) but not in MIPS.

### Wingate test: anaerobic power

**Figure 2:**
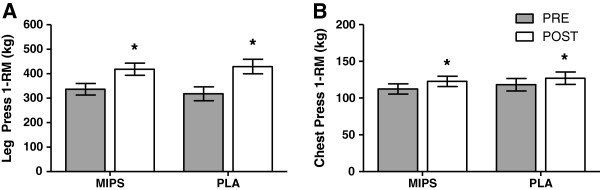
**Wingate Peak Anaerobic Power (W) before and after six weeks of resistance training and supplementation with multi-ingredient performance supplement (MIPS, n = 12) or placebo (PLA, n = 10).** There was a main time effect (p = 0.002). *Post-hoc analysis indicated a significant increase for MIPS only (p < 0.05). Bars are means ± SE.

There were no group x time interactions for any of the Wingate variables. There was a main time effect for peak anaerobic power (p = 0.001, Figure 2), relative peak anaerobic power (p = 0.001), mean anaerobic power (p = 0.007), relative mean anaerobic power (p = 0.016), and total work (p = 0.006). Post-hoc analysis revealed that the MIPS group significantly increased peak anaerobic power by 16.2% (PRE, 932.7 ± 172.5 W vs. POST, 1119.2 ± 183.8 W, p = 0.002), relative anaerobic power by 9.4% (PRE, 11.1 ± 1.7 W·kg^-1^ vs. POST, 13.1 ± 1.8 W·kg^-1^, p = 0.003), mean anaerobic power by 9.9% (PRE, 676.4 ± 145.3 W vs. POST, 751.1 ± 1.8 W, p = 0.02), and relative mean anaerobic power by 8.2% (PRE, 7.9 ± 1.0 W·kg^-1^ vs. POST, 8.8 ± 1.1 W·kg^-1^, p = 0.03) while PLA remained unchanged. There were no changes in fatigue index for either group.

### One Repetition Maximum (1RM) Strength

**Figure 3:**
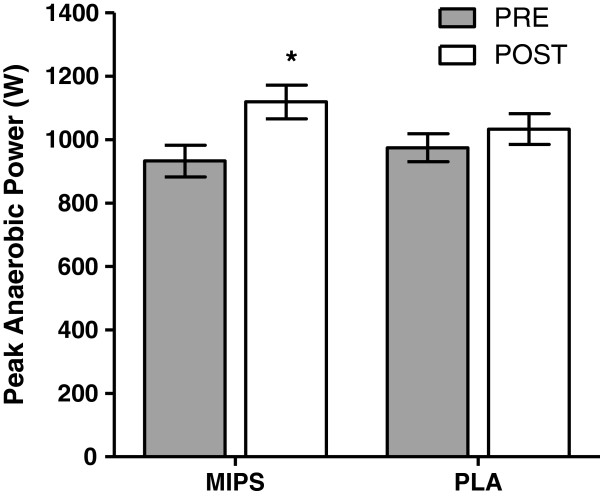
**Leg press one-repetition maximum (1RM) (A) and chest press 1RM (B).** * Indicates main time effect (p = 0.001). Before and after six weeks of resistance training and supplementation with multi- ingredient performance supplement (MIPS, n = 13) or placebo (PLA, n = 9). Bars are means ± SE.

There were no group x time interactions observed for any maximal strength variable. Time effects were noted for all 1RM measures (p = 0.001). Post-hoc analysis indicated that in LP, the MIPS group increased with RT by 19.6% (PRE, 336 ± 24 kg vs. POST, 418 ± 25 kg, p < 0.001) and the PLA group increased by 25.9% (PRE, 318 ± 28 kg vs. POST, 429 ± 29 kg, p < 0.001). For CP, MIPS increased 8.4% (PRE, 112 ± 7 kg vs. POST, 123 ± 7 kg, p = 0.001) and the PLA group increased by 7.0% (PRE, 118 ± 8 kg vs. POST, 127 ± 8 kg, p = 0.001) (See Figure 3).

When adjusted for individual LM (relative strength), the previously noted time effects were maintained for all 1RM measures (p = 0.001). Post-hoc analysis indicated that in LP, the MIPS group increased with training by 19.9% (PRE, 11.5 ± 2.8 vs. POST, 14.4 ± 2.6, p < 0.001) and the PLA group increased by 25.8% (PRE, 10.8 ± 1.8 vs. POST, 14.6 ± 2.2, p < 0.001). For CP, MIPS increased by 8.6% (PRE, 3.9 ± 0.8 vs. POST, 4.2 ± 0.8, p = 0.001) and the PLA group increased by 6.9% (PRE, 4.0 ± 0.5 vs. POST, 4.3 ± 0.5, p = 0.001).

### Three-day food intake

Eight participants satisfactorily completed the three-day food logs (MIPS, n = 5; PLA, n = 3). In this subset, there were no significant differences between groups in average kilocalories (MIPS, 37.6 ± 8.3 kcal/kg/day vs. PLA, 25.3 ± 5.8 kcal/kg/day, p = 0.34), protein (MIPS, 1.9 ±0.5 g/kg/day vs. PLA, 1.4 ± 0.3 g/kg/day, p = 0.56), carbohydrate (MIPS, 3.2 ± 0.7 g/kg/day vs. PLA, 2.5 ± 0.8 g/kg/day, p = 0.49), fat (MIPS, 1.8 ± 0.8 g/kg/day vs. PLA, 1.0 ± 0.9 g/kg/day, p = 0.51), or caffeine (MIPS, 2.2 ± 0.8 mg/kg/day vs. PLA, 1.9 ± 0.7 mg/kg/day, p = 0.49) consumed before or after training.

## Discussion

The objective of this study was to determine the efficacy of pre- and post-RT supplementation with MIPS on body composition, muscle strength, and power in resistance-trained men participating in a six-week periodized RT program. With this specific population, any gains in strength should be almost entirely due to physiological and hypertrophic changes to the trained muscles, rather than improvements in neuromuscular coordination. Shelmadine et al. [14] noted large increases in markers of satellite cell activation and hypertrophy, and modest increases in LM (4.8%) for their MIPS group after only four weeks in untrained men. By increasing the time course and total volume of training in the present study, we aimed to augment the opportunity for muscle growth. In addition to ingesting SHOT before exercise, our participants also consumed one serving of SYNTH immediately post-exercise and on every non-training day. This supplementation model, similar to that used by Spillane et al. [21], provided a better environment for muscle hypertrophy and recovery and supplement loading than the modality used by Shelmadine et al. [14]. Both Shelmadine et al. and Spillane et al. [14,21] allowed participants to train independently, while the present study monitored all training sessions with experienced research staff that provided form corrections and spots for free-weight lifts. The emphasis placed on consistent lifting form in this study, coupled with researcher supervision, helped ensure full participant compliance with training as well as reduced variability due to inter-subject differences or deficiencies in form.

Changes observed in body composition were perhaps the most remarkable results of the current study. MIPS increased LM by 4.7%, a degree similar to those observed in untrained males by Spillane et al. (3.5%) and Shelmadine et al. (4.8%) [14,21] and greater than that observed in trained males by Schmitz et al. (2.4%) [22]. Because there were no changes in FM, the decreased %BF observed in the MIPS group was due to increased LM and overall body mass. The PLA group made no significant changes in any body composition variable, although there were trends for improved LM. The lack of change in FM demonstrated in this study reflects the findings of other similar studies [13,14,29-31], but is at odds with popular claims made about these products. One of the proprietary blends listed on the SHOT label contains 376 mg of a combination of caffeine, β-phenylethlylamine HCL, *hordeum vulgare* bud, and L-tyrosine, and is marketed in SHOT and in other similar products as a “fat burning” component. However, because participants were instructed to consume their normal dietary intake rather than being fed specific meals with specific caloric restrictions, we cannot draw the conclusion that SHOT and SYNTH consumption pre- and post-exercise are ineffective at reducing FM. However, it is worth noting that no changes in dietary intake were reported from baseline (week 0) to post-testing (week 6) in a subset (n = 8) of our participants, therefore, our lack of change in body mass (kg) is likely real. Perhaps more valuable to consumers, limb circumferences increased only in thigh measurements for the MIPS group, but not for the PLA group.

A significant increase in LM was measured in the MIPS group but not in the PLA group. This is in concurrence with many similar studies [13,14,29-31]. As muscle mass is one of the main determinants of strength and power [32], it is somewhat unexpected that the MIPS group did not experience greater improvements in 1RM strength, although 1RM tests may not be sensitive enough to detect the modest difference in LM improvement exhibited by the MIPS group by these trained men. Likewise, this most likely explains the lack of group x time effects in circumference measurements other than thigh. One remarkable finding of this study is that the increase noted in LM by the MIPS group in this study (+4.7%) was very similar to that of the supplement group in Shelmadine et al. (+4.7%) [14], despite the increased training status of our participants.

While the present study noted a main time effect for peak and average anaerobic power and total work performed, there were no differences between the two groups. There was, however, a strong trend (group × time effect, p = 0.06) for the MIPS group to improve peak anaerobic power. We also noted an increase in mean anaerobic power for MIPS, but not for PLA (Figure 1). These findings are similar to those of Beck et al. [13], who demonstrated significant increases in peak and mean anaerobic power following 10 weeks of RT using untrained males consuming a pre-exercise supplement containing protein, creatine, and BCAAs. The protocol used by Beck et al. called for two consecutive 30-second cycling bouts, whereas the present study only used a single bout. The differences in training duration (six weeks vs. 10 weeks), number of cycling bouts, and training status may explain why Beck et al. were able to elicit significant group x training effects while we were not.

We observed a significant (p = 0.035) time effect for resting serum testosterone to increase with chronic training, but no group x time effects were observed. This is in contrast to a study by Rankin et al. [33] which demonstrated decreases in testosterone following nine weeks of RT and supplement consumption, but in agreement with other studies linking whole body RT programs with enhanced testosterone. Coryceps sinesis, an ingredient included in the MIPS utilized for this study, is purported by supplement manufacturers to enhance testosterone levels in males. Based on our findings and those of Hsu et al. [34] who specifically looked at the effect of coryceps sinesis on testosterone in conjunction with RT, we conclude that MIPS and coryceps sinesis did not enhance resting testosterone concentrations in response to chronic exercise in the present study. In addition, in the present study no changes for either group were noted in IGF-1 or hGH. Shelmadine et al. [14] and Spillane et al. [21] reported a time effect for IGF-1, but did not observe group x time effects. With the similarity in supplementation and training protocols between Spillane et al. [21] and ours, differences in training status may explain why our participants did not exhibit detectable changes in IGF-1. Neither Shelmadine et al. [14] nor Spillane et al. [21] investigated changes in testosterone or hGH. Our observation of no change in hGH with RT is in agreement with Kraemer et al. [35], who measured basal hGH following three, six, and eight weeks of resistance training in untrained males. It is possible that due to the training status of these men, changes in these anabolic hormones may have been blunted.

It was also expected that the inclusion of beta alanine in MIPS would yield improvements in fatigue index through the lactate buffering effects of carnosine. Instead, we found no significant time or group × time effect for fatigue index, in contrast to the findings of others [5,36,37]. Hoffman et al. noted improvements in fatigue index following 30 days of beta alanine supplementation in American football players during offseason training [5]. Some of this discrepancy may be explained by beta alanine dosages. In studies that have demonstrated improvements in performance, beta alanine dosages tend to range from 4.8 to 6.0 g·day^-1^[29,36,38,39]. Unfortunately, the MIPS in the present study included beta alanine as part of a proprietary blend, rather than labeling it independently and, therefore, we do not know the true concentration of beta alanine in the product. We can only speculate, therefore, that our MIPS group may have been consuming less than the 4.8 g/day that has been shown to elicit training enhancements.

The present study demonstrated a significant effect of time for both CP and LP strength in both groups; however, there was no group x time effect. Shelmadine et al. [14] also noted a training effect for both groups in CP and LP following 28 days of RT with SHOT supplementation before RT for 28 days. They noted that the SHOT supplemented group improved CP significantly more than the placebo group (18.4% vs. 8.8%, respectively, p = 0.003)[14]. In contrast to Shelmadine et al., Beck et al. [13] reported no differences in training-induced enhancements in CP or LP between a creatine-protein supplement group and placebo groups in their 10-week RT study [13]. Cribb et al. were able to elicit 1RM group × time effects in trained males following 10 weeks of RT and consumption of whey protein [40] or whey protein and creatine [41]. With so much conflicting evidence and confounding variables, it is difficult to draw conclusions about the effectiveness of MIPS on 1RM strength in trained males. It is worth noting, however, that in all of these studies the supplement group increased LM significantly more than the placebo.

Isokinetic leg exercise results were mixed. There appeared to be a pattern for both groups to improve strength and power during flexion but to make little improvement or even decrease performance in extension, as was the case with 30°sec^-1^ extension in the MIPS group. However, the MIPS group did exhibit trends (p = 0.054) for improvements in some 60°sec^-1^ extension variables. Training specificity is one explanation for these data; our training program included seated hamstring curls, but not knee extensions. Thus, each participant spent six weeks without doing seated extension types of exercise (they participated in leg press and lunge exercises instead). Little investigation has been conducted into the effect of MIPS and RT on isokinetic strength. These results are surprising as single-supplement [29,36,42-44] and training-alone [45,46] studies have demonstrated modest increases in isokinetic performance following RT.

Results of the isometric tests are particularly puzzling, as the MIPS group made no improvements while the PLA group improved in several measures during flexion. This is in contrast to other studies using supplement combined with training [47,48] and correlations of muscle mass and isometric force production [32]. There are a few possible explanations for these findings. Neither group in the present study performed isometric exercise as part of training. Thus, training specificity may have played a role in the lack of findings; however, this would have applied to both groups equally. Moreover, isometric exercise performance is somewhat sensitive to innate muscle fiber type distribution [49], which was not tested or controlled for in this investigation.

We observed no differences in volume (weight lifted × repetitions x sets) lifted for any exercise over the course of the training period. This was in contrast to common findings of other supplement plus training studies involving caffeine [12,50], beta alanine [5,9], and creatine [9], but not all studies [4]. The lack of difference between groups in training volume may have been a result of our study design rather than supplement effects. All participants were instructed that the goal of every set should be failure and they were to achieve this by selecting weights that caused them to fail at a specific number of repetitions (10 for weeks one and two, six for weeks three and four, and four for weeks five and six). The number of repetitions was controlled in order to facilitate the periodized training goals. If participants lifted to failure on every set, differences in training volume may have been evident. On the other hand, eliminating training volume as a variable leaves manipulation of hypertrophic pathways by the supplement ingredients as the most probable explanation for increased LM in MIPS but not for PLA. In addition, all of the participants had performed the required exercises in past workouts prior to beginning the study. The participants were also familiar with overloading the muscles with periodized training. However, we did not survey or record the degree to which the study routine was similar to or different from the participant’s regular workout program.

## Conclusions

Consumption of MIPS before and after RT during the course of a periodized six-week RT program resulted in significant improvements in LM in trained males, whereas the consumption of an isocaloric PLA did not. At the dosages consumed and with the specific population in this study, MIPS consumption did not appear to offer advantages in measures of absolute or relative muscle strength, but it did elicit gains in anaerobic power. Continued investigation of these or similar products is warranted as questions about the influence of performance supplements on volitional training volume should be answered. Additionally, future research should investigate MIPS use in populations that include both women and older populations and incorporate exercise modalities that extend beyond traditional resistance training.

## Abbreviations

%BF: Percent body fat; 1-RM: One repetition maximum; BCAA: Branched chain amino acid; DXA: Dual X-ray Absorptiometry; FFM: Fat free mass; FM: Fat mass; hGH: Human Growth hormone; HIIT: High intensity interval training; IGF-1: Insulin-like growth factor-1; LM: Lean mass; MHC: Myosin heavy chain; MIPS: Multi-ingredient performance supplement; MVC: Maximal voluntary contraction; PLA: Placebo; RE: Resistance exercise; RT: Resistance training; SHOT: NO-Shotgun multi-ingredient performance supplement; SYNTH: NO-synthesize multi-ingredient performance supplement.

## Competing interests

This study was supported by an independent research grant and product donation from Vital Pharmaceuticals, Inc. (Davie, FL). None of the authors had financial or other interests concerning the outcomes of the investigation. The authors declare that they have no competing interests.

## Authors’ contributions

MJO conceived and designed the study, secured funding for the project, provided oversight of data collection, analysis, biochemical assays, and manuscript preparation. WKM carried out data collection, participant recruitment, exercise training, laboratory testing, and manuscript preparation. DDT carried out subject recruitment, data collection, exercise training, immunoassays, and assisted with manuscript preparation. AWK and EGW helped extensively with data collection. LBP and JSK provided assay support, and insight into drafting the study design and manuscript. All authors read and approved the final manuscript.
